# Diagnostics for priority bacterial pathogens: global gaps and research needs for curbing antimicrobial resistance in low-resource settings

**DOI:** 10.1016/j.lanmic.2026.101385

**Published:** 2026-07

**Authors:** Valeria Gigante, Maurine Murtagh, Till T Bachmann, Tamarie Rocke, Betsy W Trainor, Susan M Poutanen, Teri Roberts, Jordi Vila, Alexandra Cameron

**Affiliations:** a AMR Department, WHO, Geneva, Switzerland; b The Murtagh Group, Woodside, CA, USA; c Centre for Inflammation Research, Institute for Regeneration and Repair, University of Edinburgh, Edinburgh, UK; d Combating Antibiotic-Resistant Bacteria Biopharmaceutical Accelerator (CARB-X), Boston, MA, USA; e Sinai Health, University Health Network, University of Toronto, Toronto, ON, Canada; f Global Antibiotic Research and Development Partnership, Geneva, Switzerland; g Barcelona Institute for Global Health (ISGlobal), Hospital Clinic, University of Barcelona, Barcelona, Spain; h CIBERINFEC, Instituto Salud Carlos III, Madrid, Spain

## Abstract

Antimicrobial resistance (AMR) is a major and growing threat to global health, development, and security, with the greatest burden borne by low-income and middle-income countries (LMICs). Without urgent intervention, cumulative deaths between 2020 and 2050, attributable to AMR, are projected to be 39·1 million globally. Effective diagnostics are important to bacterial pathogen detection and antimicrobial susceptibility testing, and such diagnostics support antibiotic stewardship by reducing adverse drug events (eg, toxicity or allergy) and restricting the emergence and spread of AMR. Yet, access to appropriate diagnostics in LMICs remains constrained. This Review summarises the current landscape of commercial and pipeline bacterial in-vitro diagnostics, drawing on the updated WHO 2024 Bacterial Priority Pathogen List, the 2025 WHO diagnostic landscape analysis, and the WHO Diagnostic Initiative, which has four pillars, one of which focuses on research and innovation to improve diagnostics for AMR. We discuss phenotypic and non-phenotypic testing approaches, assess platform suitability across health system tiers in LMICs, and outline WHO priorities for the next 3–5 years to guide innovation and improve equitable access. Although multiple commercial platforms exist for bacterial identification and susceptibility or resistance testing, most platforms are infrastructure-intensive and confined to regional or reference laboratories, leaving major diagnostic gaps at primary and secondary care levels. Promising advances include rapid immunoassays and emerging molecular platforms; however, no simple, affordable, decentralised solution provides reliable identification and susceptibility or resistance testing for key bacterial priority pathogens.

## Introduction

In 2019, WHO recognised antimicrobial resistance (AMR) as a top global health priority. AMR disproportionately burdens low-income and middle-income countries (LMICs).^1^ According to the Global Burden of Disease Study 2024, cumulative deaths between 2020 and 2050 attributable to AMR would be 39·1 million in the absence of further policy interventions; an additional 169 million lives might be lost indirectly owing to complications associated with AMR.^2^^,^^3^

**Search strategy and selection criteria**

The Review methods included three components: a desktop review of publicly available data, a literature review, and expert consultations. During the desktop review, the authors (VG, MM, TTB, TR, BWT, SMP, TR, JV, AC) comprehensively assessed relevant available information, including published reports from recognised health organisations (eg, Unitaid, the Foundation for Innovative Diagnostics [FIND], and WHO), conference reports, abstracts and posters, systematic reviews, and reports of discussions with WHO staff and external experts. We also searched the websites of individual commercial diagnostic platform companies, US Food and Drug Administration, European Medicines Agency, and other stringent regulatory authorities to derive operational characteristics of the profiled devices or tests from company-supplied, developer-supplied, or publicly available information.

For the literature review, we searched Embase, Cochrane Database of Systematic Reviews, and PubMed, among others from Aug 1 to July 1, 2024. The search terms included combinations of terms such as “in vitro diagnostics,” “bacterial priority pathogens,” “bacterial diagnostics,” “antibacterial resistance,” “antimicrobial resistance,” “AST,” “sepsis,” “*Streptococcus,*” “*Enterococcus,*” “*Staphylococcus,*” “bloodstream infections,” “bacterial culture,” “lateral flow assay,” “PCR,” “NAAT,” and “MALDI-TOF,” among others. The Review focused on performance data of profiled diagnostic assays or platforms derived from independent studies that were published in peer-reviewed literature, unless otherwise noted. Peer-reviewed articles, systematic reviews, and meta-analyses published in English were included, whereas editorials, commentaries, non-English publications, and studies with no diagnostic performance data were excluded.

Expert consultations were conducted with WHO staff and members and observers of the WHO Expert Group on diagnostics for bacterial infections via targeted discussions and a virtual meeting held on Dec 4, 2024. These consultations aimed to validate findings, identify diagnostic gaps, and formulate research and development priorities. We also held discussions or semistructured interviews with developers and manufacturers of diagnostic tools to obtain additional information or clarify publicly available data.

The political declaration on AMR, adopted during the 79th UN General Assembly high-level meeting on September 2024, affirmed a global commitment to tackle AMR by 2030.^4^ The declaration emphasises the crucial role of diagnostics in ensuring appropriate antibiotic usage and pledges to close the diagnostic gap, particularly in LMICs, where insufficient access to diagnostics often results in inadvertent antibiotic misuse and rising resistance. The declaration also calls for innovation in developing affordable, rapid, and reliable diagnostic tools. The declaration recognises the role that diagnostic tests along with laboratory capacity play in AMR surveillance, which is deficient in accurate, transparent, and consistent data needed to fully quantify the AMR burden and to design appropriate treatment guidelines.

**Figure 1:**
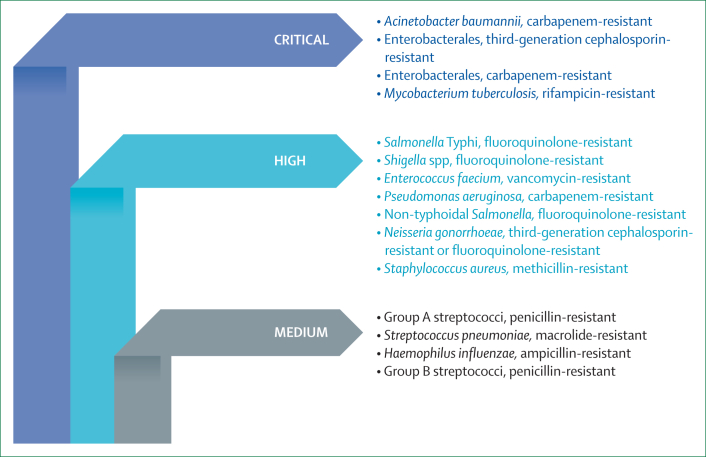
WHO 2024 bacterial priority pathogen list

In response to the increasing global health concerns regarding bacterial infections, WHO updated the bacterial priority pathogen list (BPPL) in 2024 (figure 1).^5^ Moreover, WHO conducted a landscape analysis of both currently available and pipeline-stage diagnostics to assess global preparedness for the detection and identification of these pathogens.^6^ Existing and emerging tools for pathogen detection and identification, phenotypic antimicrobial susceptibility testing (AST), and genotypic antibacterial resistance testing were identified, grouped, and compared during this analysis. The analysis further highlighted crucial gaps in the availability and accessibility of these diagnostics, particularly in LMICs, and outlined priority areas for research and development, reinforcing WHO’s commitment to fostering innovation in diagnostic technologies. In this Review, we summarise the key findings of the WHO diagnostic landscape analysis, discuss major gaps and needs, and outline research and development priorities for the next 3–5 years, which are intended to guide innovation and strengthen global access to bacterial diagnostics, with particular emphasis on LMICs.

## Scope of the Review

For this Review, we examined both commercially available and pipeline-stage in-vitro diagnostics relevant to the WHO BPPL; however, we did not consider diagnostics or platforms intended for *Mycobacterium tuberculosis* as a dedicated report was developed by Unitaid after its classification as a critical-priority pathogen in the updated WHO BPPL.^7^ Unitaid has prepared a dedicated landscape report for *Neisseria gonorrhoeae* as well.^8^ Hence, this organism, classified as a high-priority pathogen owing to its rapidly evolving resistance, spread in communities, and inadequate treatment and diagnostics options*,*^9^ was also not included in the Review. Laboratory-developed tests, in-house tests, non-in-vitro diagnostics, and diagnostics intended for use in the animal, environmental, and agricultural sectors were also excluded. We considered phenotypic and genotypic in-vitro diagnostics for pathogen detection and identification, tools for assessing AST or antibiotic resistance profiles, assays that differentiate bacterial and non-bacterial infections, and immunological biomarker tests that detect host responses (such as marker of disease severity or tests to rule out bacterial infections) at all levels of the health-care system in all settings.

## Laboratory systems in LMICs

**Figure 2:**
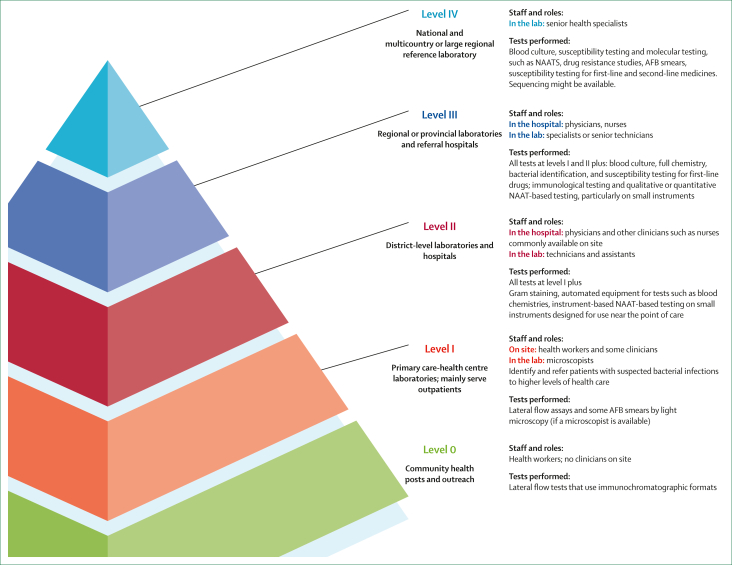
Tiered laboratory system in LMICs Laboratory systems in LMICs are often depicted as a pyramid, which illustrates that there are generally a large number of level 0 (community health posts) and level I (primary care) facilities that serve the most patients directly. However, these levels have little or no laboratory infrastructure or laboratory testing capabilities. Levels II (district laboratories and secondary care facilities), III (regional or provincial laboratories and tertiary care facilities), and IV (national reference laboratories) include a smaller number of facilities than at the lower levels. However, the higher levels are more centralised with improved laboratory infrastructure and expanded testing capabilities. In addition, national reference laboratories and some provincial laboratories might not serve patients with a broad set of consultative services but instead function as referral centres for quality assurance and training or for conducting complex tests. AFB=acid-fast bacilli. LMICs=low-income and middle-income countries. NAAT=nucleic-acid amplification test.

Understanding laboratory testing in LMICs requires consideration of the typical health-care facilities and testing services available, which are usually characterised as a tiered system (figure 2).^10^ The tiered levels of a laboratory system and the testing performed at each level might vary depending on the population served (eg, infants or adults), level of service available, physical infrastructure, electricity, water, road conditions, and the availability of trained technical personnel in-country, and these factors will vary by country. The WHO essential diagnostics list (EDL) also uses this tiered laboratory system.^11^

## Detection and identification of priority bacterial pathogens

### Traditional phenotypic methods for the detection and identification of bacterial pathogens

Phenotypic methods for the detection and identification of bacterial pathogens, which include bacterial culture, microscopic and macroscopic morphology analysis, and biochemical testing, are referred to as traditional, classic, or conventional methods. They rely on features of the organism, including cell and colony morphology and biochemical reactions, beyond its genetic make-up.

#### Bacterial culture

Phenotypic identification of bacteria relies primarily on bacterial culture, in which different types of media such as chromogenic media, are used.^12^ Although bacterial culture allows for isolation and semiquantitative analysis, it has limitations such as long incubation times (12 h–5 days), risk of contamination, and the need for specialised expertise.^13^, ^14^, ^15^ In addition, some fastidious bacteria might not grow in culture media.^13^^,^^14^

Despite the disadvantages of bacterial cultivation, the technique is still essential to definitively identify many pathogens in the BPPL. In particular, blood culture is required to identify bacterial pathogens responsible for bloodstream infections (BSIs), which are a common cause of morbidity and mortality worldwide, with an estimated fatality range of 15–30%.^16^ However, owing to the need for specialised expertise and infrastructure, bacterial culture is not performed routinely in primary or secondary care centres in LMICs.

#### Morphology

Microscopy can be performed on cultured samples or on clinical specimens. It confirms the presence of bacteria, allows detection of different organisms present in the same specimen, and helps to evaluate an organism’s clinical significance.^17^ After staining, microscopy differentiates bacteria on the basis of cell wall properties; for example, microscopy after Gram staining helps to classify Gram-positive and Gram-negative bacteria.^17^ However, the Gram stain alone is not sufficient to definitively identify bacteria.

Macroscopic (colony) morphology of bacteria can also be examined via bacterial culture. The properties of an individual bacterial colony, including its form, size, elevation, margin or border, surface, opacity (eg, glistening, opaque, or dry), and colour (pigment), can be examined. Although these characteristics provide clues on the identity of the bacteria, colony morphology alone is not sufficient for definitive identification.^17^

#### Biochemical testing

Biochemical testing of bacteria, in which the nutritional and metabolic capabilities of a bacterial isolate are analysed, is often used in combination with bacterial culture, microscopy, and morphology analysis to determine the genus and species.^18^ In general, a battery of tests is run to identify the bacteria’s metabolic profile, which is then compared with an extensive identification database to establish the identity of the specific isolate.

Conventional phenotypic methods of detection and identification of bacterial pathogens are usually performed manually. However, automated systems are available for most steps of the process. These include commercially available systems for Gram staining, inoculation and specimen processing, bacterial cultivation, biochemical testing or metabolic profiling, and automatic detection of growth on agar media.^6^ These systems can standardise the processes of phenotypic identification methods and reduce turnaround time. However, most systems are best suited for sophisticated high-throughput laboratories and are costly to implement, requiring well-trained technicians and appropriate infrastructure, including adequate space, consistent power supply, climate control, running water, laboratory consumables, access to transport for specimen collection and dispatch to laboratories, and reliable supply chains. These requirements restrict accessibility to these systems in LMICs, especially in level I and level II health-care facilities.

### Immunoassay methods for the detection and identification of bacterial pathogens

Rapid immunoassays, in which the binding of antibodies to antigens is used to identify and measure specific substances, can be used for the detection and identification of select bacterial pathogens. Most commercially available rapid immunoassays identify group A streptococci, group B streptococci, or *Streptococcus pneumoniae* and can be performed without bacterial culture. Rapid immunoassays are also available for other pathogens in the BPPL, including *Salmonella* Typhi, *Escherichia coli,* and *Shigella* spp, which often require cultured isolates.^6^

Although many of these assays require little or no ancillary equipment and can generally be used at level I or II health-care facilities in LMICs, some tests, such as rapid immunoassays that are performed after a positive culture result, are complex enough to be performed only in a laboratory setting. Most of these assays support multiplexing only to some extent. The assays are also associated with technical challenges, including potential cross-reactivity.^19^^,^^20^ Each assay should be assessed to ensure whether its sensitivity and specificity are adequate for the intended use of the test and whether its ease of use is appropriate for the intended setting.^21^

### Molecular methods for the detection and identification of bacterial pathogens

Molecular testing methods can be used to detect specific sequences of DNA and RNA. These methods have substantially changed the diagnostic landscape of bacterial and other microbiological pathogens over the past decade. In particular, nucleic-acid amplification tests (NAATs) are commonly used in clinical laboratory diagnostics. Molecular testing methods generally fall into one of the three categories—namely, hybridisation, amplification, or sequencing.^22^

#### Hybridisation methods

Hybridisation assays use labelled oligonucleotide probes to identify bacterial pathogens and are typically applied after positive blood culture results. The most commonly used hybridisation is peptide nucleic acid fluorescence in situ hybridisation (PNA-FISH), which enables rapid genus-level and species-level bacterial identification using only a fluorescence microscope, combining the speed of conventional staining with the specificity of molecular methods.^23^ PNA-FISH assays are, however, being phased out in favour of simpler, more universal PCR-based assays.

#### Amplification methods

Various commercial molecular amplification methods are available to detect and identify bacterial pathogens, among which, NAATs, specifically PCR-based assays (for which many types of detection probe formats are available), are most commonly used.^17^^,^^24^^,^^25^ Many such assays are real-time systems, and some support multiplexing. Similarly, a few commercially available platforms that use isothermal amplification techniques, such as loop-mediated isothermal amplification (LAMP) and recombinase polymerase amplification, are also available. The effectiveness of these platforms for the detection and identification of a range of pathogens in the BPPL, including enteric pathogens and those causing BSIs and lower respiratory tract infections, has been extensively validated and documented.^26^, ^27^, ^28^

Molecular methods typically yield results faster than classic phenotypic methods and can often be performed using specimens directly from sterile sites (eg, blood, serum or plasma, and cerebrospinal fluid); however, cultured specimens or positive blood culture bottles are required for almost all molecular tests for BSIs. This requirement slows the turnaround time and confines their application to laboratories in which bacteria can be cultured. In addition, bacterial load in blood is typically low (1–100 colony-forming units per mL) and often below the limits of detection of most commercial molecular assays, thus restricting the sensitivity of direct-from-blood NAATs in routine clinical practice.^13^

The table presents a non-exhaustive list of commercially available molecular test platforms that use NAAT-based assays, including PCR, hybridisation, and other methods, to identify priority bacterial pathogens. The details include sample types, species detected by the assays, and whether the platform could be used to test for antibacterial resistance.

**Table:** Molecular amplification-based products for identifying priority bacterial pathogens

	Assay method	Sample type	Species detected	Antibiotic resistance testing (yes or no)
cobas liat system (Roche Molecular Diagnostics, USA)	Nucleic acid purification and PCR	Throat swab specimens	Qualitative detection: *Streptococcus pyogenes* (group A β-haemolytic *Streptococcus* or Strep A)	No
GBS and MRSA assays for the Hologic Panther Fusion system (Hologic, USA)	Real-time TMA and PCR	GBS: enrichment broth cultures of vaginal or rectal swabs from antepartum women; MRSA: nasal swab specimens	GBS: qualitative detection of *Streptococcus agalactiae* (Group B β-haemolytic *Streptococcus* or Strep B) MRSA: qualitative detection and differentiation of *Staphylococcus aureus* and MRSA	Yes
Enteric bacterial and extended enteric bacterial panels for the BD MAX system (BD, USA)	Real-time PCR; fluorescent detection	Soft to liquid stool specimens or stool samples preserved in Cary Blair transport media	Enteric panel: *Salmonella* spp; *Campylobacter* spp (*Campylobacter jejuni* and *Campylobacter coli*); *Shigella* spp or EIEC; and *stx1* or *stx2* genes (found in STEC); Extended enteric panel: *Plesiomonas shigelloides; Vibrio (Vibrio vulnificus, Vibrio parahaemolyticus, and Vibrio cholerae);* LT and ST genes in ETEC; and *Yersinia enterocolitica*	No
Stool bacterial pathogens panel, GBS test, Shigatoxin direct test, and Staph ID/R BC panel for Great Basin Analyser system (VELA Diagnostics, Singapore)	Hot-start PCR and hybridisation probes	Stool bacterial pathogen panel: stool; GBS: vaginal or rectal swabs from antepartum women; Shiga toxin direct: stool; Staph ID/R BC panel: positive BC	Stool bacterial pathogen panel: *Campylobacter* (*C coli* or *C jejuni*), *Salmonella*, *stx1* and *stx2* genes, *Escherichiacoli* (*E coli*) serotype 0157, and *Shigella;* GBS panel: GBS; Shiga toxin direct: *stx1* and *stx2* genes and *E coli* O157; Staph ID/R BC panel: *Staphylococcus aureus*, *Staphylococcus lugdunensis*, *Staphylococcus* spp identified to the genus level, and detection of the *mecA* gene	Yes
Solana GAS assay, Solana Strep Complete assay, and Solana GBS Assay for Solana Molecular testing platform (QuidelOrtho, USA)	Helicase-dependent amplification (isothermal) with fluorescence labelled probes	GAS and Strep complete assays: throat swab specimens; GBS assay: vaginal or rectal swabs from antepartum women after 18–24 h of incubation	GAS assay: group A β-haemolytic *Streptococcus* (*S pyogenes*); Strep complete assay: *S pyogenes* (group A β-haemolytic *Streptococcus*) and *Streptococcus dysgalactiae* (pyogenic group C and G β-haemolytic *Streptococcus*); GBS assay: group β-haemolytic *Streptococcus*	No
RTI and MRSA/SA assay for the Vivalytic Analyzer (Bosch Healthcare Solutions, Germany)	Endpoint PCR, quantitative real-time PCR, melting curve analysis, and microarray technology	RTI: single sputum, lavage, or nasopharyngeal samples; MRSA/SA: nasopharyngeal or oropharyngeal swabs	RTI: *Bordetella parapertussis, Bordetella pertussis, Chlamydophila pneumoniae, Haemophilus influenzae, Legionella pneumophila, Moraxella catarrhalis, Mycoplasma pneumoniae,* and *Streptococcus pneumoniae* MRSA/SA: MRSA and meticillin-resistant coagulase-negative pneumoniae and *S pneumonia*	Yes
QIAstat-Dx gastrointestinal panel 2 for QIAstat-Dx analysers (QIAGEN NV, Germany)	Real-time PCR	Stool samples preserved in Cary Blair transport media	Qualitative detection and identification of *Shigella* or EIEC, EPEC, ETEC LT or ST, STEC *stx1* or *stx2* (including specific identification of *E coli* O157 serogroup within STEC), and *Salmonella* spp and other enteropathogens	No
xTAG gastrointestinal pathogen panel for MAGPIX (Luminex, USA, a DiaSorin company)	Multiplex RT-PCR with Luminex’s proprietary universal tag sorting system	Human stool specimens	Eleven bacterial pathogens, including *E coli*, enterotoxigenic *E coli*, *Salmonella* spp, Shiga-like toxin-producing *E coli stx1/stx2*, and *Shigella* spp	No
Simplexa group A Strep direct and Simplexa GBS direct assays for LIAISON MDX system (Luminex, USA, a DiaSorin company, Italy)	Multiplex RT-PCR with Luminex’s proprietary universal tag sorting system	Simplexa group A Strep direct: human stool; Simplexa GBS direct: vaginal or rectal specimen swabs enriched in Lim broth for 18–24 h	Qualitative detection of group A *Streptococcus* (Simplexa group A Strep direct) or group B *Streptococcus* (Simplexa GBS direct)	No
ID NOW Strep A 2 test and ID NOW platform (Abbott, USA)	Proprietary iNAAT technology	Strep A 2 assay: throat swabs	Qualitative detection of group A *Streptococcus*	No
LSL-HUTI (Llusern Scientific-Human Urinary Tract Infections) Panel Lodestar DX (Llusern Scientific, UK)	Real-time LAMP technology	Urine	Qualitative detection of pathogens causing urinary tract infections, including *E coli*, *Klebsiella pneumoniae*, *Enterococcus or S aureus*, *Proteus mirabilis*, *Pseudomonas aeruginosa,* and *Staphylococcus saprophyticus*	No
SepsiTest-UMD (Molzym Molecular Diagnostics, Germany)	RT-PCR followed by Sanger sequencing and Basic Local Alignment Search Tool (BLAST) analysis for pathogen identification	Potassium EDTA-treated or citrate-treated whole blood; BC; cerebrospinal fluid, sputum, and nasal swabs, among other specimen types	Qualitative detection of more than 200 genera of bacteria	No
BC-GP and BC-GN for the Verigene system (DiaSorin, Italy, formerly Luminex [USA])	Molecular hybridisation assay that can be used to simultaneously detect pathogens and markers using bacterial-specific DNA in a microarray format (NanoGrid Technology)	Positive BC	BC-GP: Qualitative identification of *Staphylococcus aureus*, *Streptococcus pneumoniae*, group A *Streptococci*, group B *Streptococci,* and *Enterococcus faecium*; BC-GN: *Acinetobacter* spp, *Enterobacter* spp, *E coli*, *K pneumoniae*, and *P aeruginosa,* but without distinguishing *E coli* from *Shigella* spp; BC-GP: antibiotic resistance testing: detection of *mecA* resistance marker for inferring *mecA*-mediated meticillin resistance and *vanA* and *vanB* resistance markers for inferring *vanA*-mediated or *vanB-*mediated vancomycin resistance; BC-GN: CTX-M (*bla*_*CTX-M*_), KPC (*bla*_*KPC*_), NDM (*bla*_*NDM*_), VIM (*bla*_*VIM*_), IMP (*bla*_*IMP*_), and OXA (*bla*_*OXA*_)	Yes
BioFire Film Array (bioMérieux, France)	Nested, multiplex PCR with melt curve analysis	BCID2 (BC identification) panel: positive BC broth sample; PN (pneumonia) and PN plus panels: sputum or bronchoalveolar (BAL) fluid; GI (gastrointestinal) panel: stool; RP (respiratory) panel: nasopharyngeal swab; ME (meningitis/encephalitis) panel: cerebrospinal fluid	Qualitative identification, except for the PN panel, which can be used for semiquantitative identification of 15 bacterial pathogens; BCID2: 26 Gram-positive and Gram-negative bacteria and ten antimicrobial resistance genes commonly associated with blood-stream infections; PN and PN plus: 18 bacteria and seven markers of antimicrobial resistance; GI: *Campylobacter (C jejuni* or *C coli* or *Campylobacter upsaliensis*), *Salmonella*, and several diarrhoeagenic *E coli* or *Shigella* pathotypes; RP: three bacterial pathogens—namely, *B pertussis*, *C pneumoniae*, and *M pneumoniae*ME: six bacteria—namely, *E coli* K1, *H influenzae*, *Listeria monocytogenes*, *Neisseria meningitidis* (encapsulated), *S agalactiae*, and *S pneumoniae*	Yes
BIOFIRE SPOTFIRE System (bioMérieux, France)	Nested, multiplex PCR with melt curve analysis	Nasopharyngeal swab or throat swab in transport media	R/ST (respiratory/sore throat) panel: detects and identifies nucleic acids from: *B parapertussis*, *B pertussis*, *C pneumoniae*, *M pneumoniae*, *S dysgalactiae* (group C/G Streptococcus), and *S pyogenes* (group A *Streptococcus*); R/ST panel mini: identifies *S pyogenes* (group A *Streptococcus*)	No
GeneXpert System (Cepheid, USA)	Real-time PCR and fluorogenic target-specific hybridisation	Xpert MRSA/SA BC: positive BC from BC bottles; MRSA/SA SSTI (soft tissue infections): skin and soft tissue swabs; MRSA/SA Nasal Complete: nasal swabs; MRSA NxG (next generation) assay: nasal swabs *vanA/vanB*: stool or rectal swab; Carba-R (carbapenem resistance) assay: rectal or perirectal swab	Qualitative detection and identification of bacterial infections: Xpert MRSA/SA BC; MRSA/SA SSTI; MRSA/SA Nasal Complete; and MRSA NxG; Antibacterial resistance testing: *vanA* assay (detects *vanA* gene), *vanA/vanB* assay (detects *vanA* and *vanB* genes); and Carba-R assay detects and differentiates *bla*_KPC_, *bla*_NDM_, *bla*_VIM_, *bla*_OXA-48_, and *bla*_IMP_ gene sequences associated with carbapenem nonsusceptibility) Additional qualitative assays for detection of bacterial pathogens only: Xpert Strep A, Xpert Xpress GBS assay, and Xpert GBS LB XC assay	Yes
GenoType assays and FluoroType assays and system (Bruker-Hain Diagnostics, Germany)	GenoType assays: DNA strip technology using multiplex amplification with biotinylated primers followed by hybridisation to membrane bound probes; FluoroType assays: LATE-PCR combined with fluorescence lights on or lights off probes	GenoType BC Gram-negative and Gram-positive test kits: positive BACTEC BC bottles; GenoType MRSA: cultured material; FluoroType MRSA assay: swab specimens from the nose, throat, skin, or wound; FluoroType *Enterococcus* assay: culture; FluoroType LiquidArray gastrointestinal assay: faecal/stool samples	Qualitative identification and antibiotic resistance testing: GenoType BC Gram-positive kit: identifies 17 specimens of Gram-positive cocci, including: *Staphylococcus aureus*, *E faecium,* and *Streptococcus pneumoniae*, along with the detection of *mecA* and *van* genes; GenoType MRSA kit: direct detection of MRSA from cultured material; FluoroType MRSA assay: direct detection of MRSA; Qualitative identification only: GenoType BC Gram-negative kit identifies 15 specimens of Gram-negative bacilli, including *E coli*, *Enterobacter* spp (*Enterobacter aerogenes*, *Enterobacter cloacae*, and *Enterobacter sakazakii*), *K pneumoniae*, *P aeruginosa*, and *Acinetobacter baumannii;* GenoType *Enterococcus* assay identifies *Enterococcus* spp and differentiates among *Enterococcus faecalis*, *E faecium*, *Enterococcus casseliflavus,* and *Enterococcus gallinarum*; also identifies vancomycin resistance genes; FluoroType LiquidArray gastrointestinal assay identifies a wide range of bacterial pathogens and toxins, including *Campylobacter* spp, *Clostridioides difficile*, *Salmonella* spp, and diarrhoeagenic *E coli/Shigella* spp, including O157, EHEC, EIEC, and STEC	Yes
cobas eplex BCID (BC identification) panels and system (GenMark Diagnostics, USA, a subsidiary of Roche Diagnostics)	Multiplex PCR or RT-PCR or hybridisation or electrochemical detection	Positive BC; Gram-stain required	cobas eplex BCID-GP (Gram- positive): qualitative detection and identification of *E faecalis*, *E faecium*, *Staphylococcus* spp, *Staphylococcus aureus*, *S agalactiae*, *Streptococcus pneumoniae,* and *S pyogenes,* and resistance markers *mecA*, *mecC*, *vanA*, and *vanB;* cobas eplex BCID-GN (Gram-negative) panel: qualitative detection and identification of *A baumannii*, *Enterobacter* (non-*cloacae* complex and *cloacae* complex), *H influenzae*, *K pneumoniae*, *P aeruginosa*, and *Salmonella*, along with resistance markers including carbapenem-resistant enterobacteriaceae (CRE) (*blaKPC*, *blaVIM*, *blaNDM*, *blaIMP*, and *bla*_*OXA*_) and ESBL (*bla*_*CTX-M*_)	Yes
Magicplex Sepsis Realtime Test (Seegene, South Korea)	Multiplex real-time PCR	EDTA whole blood	Semiquantitative detection of bacteria including *Streptococcus pneumoniae*, *E faecium*, *Staphylococcus aureus*, *P aeruginosa*, *A baumannii*, *Salmonella* Typhi, *K pneumoniae*, and *E coli*; Antibacterial resistance testing: detection of *vanA*, *vanB*, and *mecA*; Additional Seegene assays—namely, Seeplex, Anyplex, and Allplex, are also available and include specific drug resistance tests	Yes
ELITe MGB kits and panels (ELITechGroup Solutions, France)	Real-time qualitative or quantitative PCR	MRSA: nasal swabs and BC; CRE: rectal swabs and BC; ESBL: rectal swabs and BC; Colistin-R: rectal swabs; Meningitis: whole blood and cerebrospinal fluid	MRSA: qualitative detection and identification of *S aureus* and MRSA (in regions with *mecA-*specific and *mecC-*specific genes); Antibiotic resistance testing only using MGB kits: CRE: resistance genes of Enterobacterales (*bla*_*KPC*_, *bla*_*NDM*_, *bla*_*VIM*_, *bla*_*IMP*_, and *bla*_*OXA-48*_); ESBL: ESBL genes of Enterobacterales (*CTX-M 1/9, 14, 15* groups); Colistin-R: *mcr-1* and *mcr-2* genes; Qualitative identification: Meningitis bacterial kit detects *N meningitidis, S pneumoniae*, and *H influenzae*	Yes
Novodiag system (Hologic, USA [formerly, Mobidiag, Finland])	qPCR: targeted approach (low- plex) and microarray: multiplex approach (high-plex)	Bacterial GE+ assay: stool samples without preservatives or in FecaSwab; CarbaR+ assay: rectal swabs and pure cultures	Bacterial GE+ assay: qualitative detection of most relevant bacteria responsible for diarrhoea, including *C coli*, *C jejuni*, *Clostridioides difficile TcdB*, *Salmonella* spp, and *Shigella* spp; CarbaR+ assay: qualitative detection of bacteria resistant to carbapenem and colistin antibiotics, including the *mcr-1* gene	Yes
GenomEra CDX System (Uniogen Oy, Finland)	Multiblock thermal cycling technology that enables rapid qPCR, RT-qPCR (quantitative reverse transcription PCR), and homogenous endpoint PCR	GBS test: vaginal or rectal swab specimens in Copan eSwab; MRSA/SA AC (positive BC) test: positive BC; MRSA/SA multiswab test: swab specimens pooled in liquid medium; *S pneumoniae* test: positive BC or equivalent liquid bacteria cultures	GBS test: qualitative detection and identification of antepartum and intrapartum colonisation of GBS and *S agalactiae* in pregnant women; MRSA/SA AC test: detects and differentiates MRSA and meticillin-resistant *S aureus*; detects SA-specific DNA and *mecA* and *mecC;* MRSA SA multiswab test: simultaneously screens multiple body sites (nose, throat, groin/perineum) for MRSA colonisation; *S pneumoniae* test: qualitative detection of pneumococci only	Yes
Revogene (GenePOC, Canada, which is a Meridian Bioscience Company, USA, and a subsidiary of SD Biosensor, South Korea)	Fluorescence-based real-time PCR	Revogene Group A Strep assay: throat swab specimens; Revogene GBS LB assay: vaginal or rectal specimen; swabs in LB; Revogene Carba C: blood agar or MacConkey agar	Revogene Group A Strep: qualitative detection of *S pyogenes*; Revogene GBS LB assay: qualitative detection of GBS DNA (*cfb* [compliment factor B] gene sequence); Revogene Carba C: detects CRE from isolated colonies of Enterobacterales*, A baumannii*, or *P aeruginosa*	Yes
Molecular Mouse System (Alifax, Italy)	Multiplex real-time PCR; lab-on-chip technology	MM Sepsis panel: DNA extracted from positive BC (validated by an extraction method implemented using the M-smasher kit, [Alifax, Italy]); Gram staining recommended	Combined identification and antibacterial resistance testing: MM POS STAPH ID: identifies Gram-positive Staphylococcus: *S aureus*, *Staphylococcus epidermidis*, *Staphylococcus* spp, *S lugdunensis*, and more; also identifies resistance genes *mecA*, *mecC*, SCCmec-*orfX*, *vanA*, and *vanB* MM GRAM POS NO STAPH: identifies multiple bacteria, including *Enterococcus* spp, *E faecalis*, *E faecium*, *S agalactiae*, *S pneumoniae,* and *S pyogenes*; also identifies the resistance genes *vanA, vanB, vanC1,* and *vanC2* or *vanC3* Identification only: MM GRAM NEG ID: identifies multiple Gram-negative bacteria, including *K pneumoniae*, *Klebsiella aerogenes*, *E coli* or *Shigella* spp, *Salmonella* Typhi, Enterobacterales, *P aeruginosa*, *H influenzae*, *A baumannii*, and *N meningitidis* Antibiotic resistance testing only*:* MM GRAM NEG RES: identifies the following resistance genes: Carba NDM (New Delhi metallo-β-lactamase) (bla_NDM_), bla_KPC_, bla_VIM_, bla_OXA48_, bla_OXA23_ bla_IMP_; ESBL genes, including *CTX-M 1/9, CTX-M 2/8*, SHV (all), SHV (ESBL); the AmpC gene *CMY 2*; and the colistin resistance genes *mcr-1* and *mcr-2*	Yes

BC=blood culture. CRE=carbapenem-resistant enterobacteriaceae. CTX-M=CefoTaXime-Munich enzymes. EDTA=edetic acid. EHEC=enterohaemorrhagic *Escherichia coli*. EIEC=enteroinvasive *Escherichia coli*. EPEC=enteropathogenic *E coli*. ESBL=extended spectrum β-lactamase. ETEC=enterotoxigenic *Escherichia coli*. GBS=group B *Streptococcus*. LAMP=loop-mediated isothermal amplification. LATE=linear after the exponential. LB=Lim broth. LT=heat-labile enterotoxin. MM=molecular mouse. MRSA=meticillin-resistant *Staphylococcus aureus*. NAAT=nucleic-acid amplification test. RTI=respiratory tract infection. SSTI=skin and soft tissue infections. ST=heat-stable enterotoxin. STEC=Shiga-like toxin-producing *E coli*. *stx*=Shiga toxin. TMA=transcription mediated amplification.

As presented in the table, many commercially available molecular platforms are automated and have single or multiplex assays to detect bacterial pathogens; many of them also have separate automated instruments to perform sample preparation and nucleic acid extraction.^6^ The assays are standardised and available as quality-assured kits. However, most of these platforms are best suited for use in sophisticated laboratories with highly trained staff, and high throughput is needed to balance their costs. As such, the platforms are not well designed for use in low-resource settings, including LMICs, and might not be cost-effective.

#### Sequencing

In addition to molecular hybridisation and amplification testing methods, sequence analyses of genes or the whole genomes of pathogens are used in microbiology laboratories, primarily for research and surveillance. Sequencing methods can be used to identify bacterial pathogens that cannot be cultured. These methods can also be used to detect genetic variations in evolving pathogens that cannot be captured by targeted molecular testing.^22^

Next-generation sequencing (NGS) has largely replaced previous methods, such as Sanger sequencing, and is being performed primarily for whole-genome sequencing (WGS) or metagenomic NGS (mNGS). Both WGS and mNGS can be used to identify bacterial pathogens or perform antibacterial resistance testing.^29^^,^^30^

NGS enables genome-wide sequencing in a single run. Although NGS does not require target-specific primers, it does require the preparation of libraries in which fragments of DNA or RNA are fused to adaptors and barcodes to distinguish the DNA of the sequenced isolate after sequencing.^22^ Data analysis following sequencing remains a challenge with NGS, because it requires bioinformatics skills and computational resources to analyse large datasets.

Commercial NGS technologies currently being explored for use in clinical microbiology are either second-generation (short-read) or third-generation (long-read) sequencing.^29^, ^30^, ^31^ These technologies are implemented in two primary ways: WGS of organisms enhanced after initial bacterial culture; or identifying potential pathogenic organisms directly from clinical specimens through either targeted NGS or mNGS, thus bypassing bacterial cultivation. An advantage of third-generation sequencing is that it can be used to sequence individual DNA molecules without amplification.^29^^,^^30^ Third-generation sequencing can also be used to identify multiple pathogens, including those that are difficult to be cultured.^29^, ^30^, ^31^

Although several second-generation and third-generation WGS platforms are commonly used in sophisticated laboratories, they are used primarily for research purposes. Nevertheless, mNGS platforms approved for clinical use to identify bacterial pathogens are commercially available.

A barrier to the use of sequencing platforms is the high cost of the equipment. Other barriers include limited access to testing, the need for highly specialised expertise (especially in bioinformatics), long turnaround times, and absence of standardisation. These factors deter the use of WGS and mNGS in low-resource settings, in particular LMICs, typically restricting their use to national or regional reference laboratories.

### Other methods for bacterial pathogen identification

In addition to the phenotypic and molecular methods of bacterial pathogen identification, additional techniques are available.

#### Mass spectrometry

Mass spectrometry is an analytical technique in which chemical compounds are ionised into charged molecules, and the ratio of their mass to charge (m/z) is measured.^32^ Matrix-assisted laser desorption ionisation-time of flight mass spectrometry (MALDI-TOF MS) is the most commonly used mass spectrometry method to identify bacterial species in large clinical laboratories.^33^ After bacterial cultivation and pathogen isolation, MALDI-TOF MS is used to ionise the abundant pathogen proteins and generate a characteristic mass spectrum profile, which is then compared against a large database of reference spectra for any given organism. MALDI-TOF MS can also be used for identifying bacteria from cultures of various specimen types to detect pathogens causing bacteraemia, urinary tract infections, respiratory tract infections, or enteric infections.^33^^,^^34^

Two commercially available MALDI-TOF MS systems are widely used. Both these systems are highly automated, allowing for high throughput and speed, after bacterial culture.^6^ The turnaround time for bacterial identification using MALDI-TOF MS is typically less than that for conventional identification methods, by at least one working day.^32^ However, the high cost of equipment and the need for highly trained personnel and complex infrastructure, including stable electricity, impede its accessibility in LMICs.

#### Attenuated total reflection–Fourier transform infrared spectroscopy

Attenuated total reflection–Fourier transform infrared (ATR–FTIR) spectroscopy can be used for the identification of pathogens, including bacterial pathogens. The technology is based on passing infrared radiation through a sample, in which some radiation is absorbed, resulting in a molecular vibration or fingerprint of the sample. Following culture, ATR–FTIR spectroscopy provides rapid and label-free detection of pathogens with minimal sample preparation. It has lower operating costs than MALDI-TOF MS or PCR-based methods and has the potential to be used in portable, compact instruments suitable for use in low-resource settings. However, ATR-FTIR has significant limitations when detecting low bacterial loads and requires complex data analysis expertise and access to high-quality, comprehensive spectral libraries.^35^

Although interest in ATR–FTIR spectroscopy is growing, this technique is not widely used in clinical microbiology laboratories and has not undergone extensive validation. The method is currently being used in only one commercial diagnostic product.^6^

#### Diagnostics for AST and antibacterial resistance testing of bacterial pathogens

To guide appropriate antibiotic treatment following pathogen identification, an AST profile of the microorganism should be generated to confirm susceptibility to empirical antimicrobial agents or identify non-susceptibility.

Among phenotypic methods for AST, culture-based AST remains the most widely used approach for bacterial susceptibility profiling and can be performed manually or using rapid, growth-dependent automated systems. The method involves co-incubating a bacterial isolate with an antimicrobial agent to assess its effects on growth or viability. Conventional disc diffusion can be used to infer susceptibility from the diameter of the growth inhibition zone around the disc and is interpreted based on established breakpoints.^17^^,^^36^ The minimum inhibitory concentration, estimated using broth or agar dilution or the gradient diffusion technique, represents the lowest antimicrobial concentration that prevents visible microbial growth and provides a quantitative measure of antimicrobial susceptibility for guiding therapy and surveillance.^17^^,^^36^, ^37^, ^38^

AST methods and interpretive criteria are primarily standardised by the Clinical and Laboratory Standards Institute (CLSI) and the European Committee on Antimicrobial Susceptibility Testing (EUCAST), which issue globally used guidelines for testing procedures and clinical breakpoints.^39^^,^^40^ For routine laboratory use, CLSI and EUCAST recognise three main phenotypic AST methods: broth microdilution, disc diffusion (Kirby–Bauer), and antimicrobial gradient diffusion. Among these methods, Kirby–Bauer disc diffusion is a standardised method used to test common, rapidly growing bacterial pathogens.^41^ This method provides qualitative results only and does not generate quantitative minimum inhibitory concentration values.^41^ Nonetheless, because of its ease of use, low cost, and applicability to many bacterial species and antibiotics, it remains the most widely used AST method in clinical microbiology laboratories and is suitable for use in LMICs.^36^^,^^42^

Although reliable, manual phenotypic AST is labour-intensive and slow, as it requires pure cultures and extended incubation (approximately 16 h or more), often resulting in reporting delays of 2–3 days after specimen collection. Semiautomated and fully automated platforms are commercially available and can help to reduce turnaround time and laboratory workload.

At least one semiautomated and several fully automated phenotypic platforms are widely used in high-income countries for combined bacterial identification and AST. Such platforms offer streamlined workflows, quantitative results, and relative ease of operation.^41^, ^42^, ^43^, ^44^ However, these systems are often unavailable in LMICs and, similar to manual AST, require cultured isolates; with susceptibility inferred from organism growth, typically measured based on turbidity in broth or growth inhibition on agar. Consequently, these systems remain constrained by the time required for culture and testing and by reduced diagnostic yield when prior antimicrobial exposure suppresses organism growth.^33^^,^^36^ Quicker, culture-independent methods are therefore needed.

Non-phenotypic or genotypic methods can be used to detect genetic determinants associated with AMR because many phenotypic resistance mechanisms are encoded by specific genes or mutations.^17^ These methods include PCR, LAMP, DNA microarrays, and NGS. In addition, several lateral flow immunoassays detect resistance gene products, typically resistance enzymes, rather than the underlying genes.

NAAT-based methods, particularly PCR, are the most widely adopted genotypic tools for pathogen identification and resistance characterisation. Multiplex PCR enables the simultaneous detection of multiple AMR genes, and commercial systems increasingly provide assays that identify pathogens and resistance genes directly from positive blood cultures or, in some cases, other clinical specimens (eg, respiratory samples or urine and urogenital swabs).

As shown in the table, numerous molecular platforms are commercially available for combined bacterial pathogen identification and antibacterial resistance testing. Almost all platforms require bacterial cultivation for the detection and identification of BSIs, increasing the turnaround time. Some platforms can be used to identify multiple genes that directly confer antibiotic resistance but cannot identify pathogens.

Although faster than phenotypic methods, these genotypic approaches to antibacterial resistance testing have important limitations. The presence of a resistant gene or mutation does not always result in phenotypic resistance, as gene expression and other mechanisms can influence outcomes.^33^^,^^34^ In addition, these methods are relatively costly, and all but a few platforms are suitable only for well resourced laboratories, thus restricting their utility in LMICs.^33^^,^^34^ Importantly, the genotypic methods do not replace culture-based identification and susceptibility testing.

## Emerging, pipeline-stage diagnostics for bacterial pathogen identification and AST or antibacterial resistance testing

In addition to commercial phenotypic and non-phenotypic methods for bacterial identification and AST or antibacterial resistance testing, a diagnostic pipeline is emerging. Platforms under development use molecular technologies, often integrated with WGS, machine learning, or artificial intelligence. Although many are designed for single assays, some offer multiplexing capability.^6^ Other emerging platforms incorporate digital imaging and artificial intelligence, which might enable automated reading of Gram smears and culture plates and support remote consultation.

Some of these systems are expected to be smaller and simpler to use than conventional platforms designed for use in large laboratories and might ultimately be suitable for use down to level II settings in LMICs. Some platforms are being designed to detect and identify bacterial pathogens, including those causing BSIs, directly from whole blood, so as to address a gap in existing technologies. However, detection and identification of bacteria directly from whole blood with sensitivity and specificity equivalent to blood culture remain challenging for several reasons, including the typically low bacterial load in blood and the presence of host-derived interfering substances.^45^

## Host response and biomarker detection assays

The diagnostic systems reviewed above focus on methods for specific bacterial pathogen detection and identification. Other rapid, easy-to-use diagnostics that do not directly detect pathogens might also help address antibacterial resistance and be suitable for use in level I and II settings in LMICs. These diagnostics include host immune response assays, such as tests that detect blood-based biomarkers. Some of these assays are commercially available, and others are in the development pipeline.

Host-derived immune response biomarkers of infection include C-reactive protein and procalcitonin.^46^ C-reactive protein is a non-specific inflammation-related protein produced in the liver and regulated by plasma interleukin-6. C-reactive protein levels increase during bacterial infections, including tuberculosis, in postoperative states, and during tissue injury.^47^ Procalcitonin is a glycoprotein with no hormonal activity and, similar to C-reactive protein, can help to distinguish between bacterial and viral infections. Measurements of procalcitonin and C-reactive protein might guide appropriate antibiotic use, particularly by ruling out serious bacterial infection and supporting antibiotic stewardship.^48^

C-reactive protein assays are used to guide antibiotic treatment.^49^^,^^50^ Most studies on C-reactive protein have reported statistically significant differences in C-reactive protein levels between people with bacterial infections and those with non-bacterial infections.^51^^,^^52^ A Cochrane review found that point-of-care C-reactive protein testing can considerably reduce antibiotic prescription for acute respiratory infections.^47^ However, C-reactive protein testing is not a substitute for a proper clinical examination.^53^

Procalcitonin levels are generally higher during severe bacterial infections. Procalcitonin testing might support clinical decision making on initiating or discontinuing antibiotic treatment across a range of infections and settings, including primary care, emergency departments, and hospital wards.^54^^,^^55^ Procalcitonin is more specific than other inflammatory markers, including C-reactive protein, for bacterial infection.^56^ According to a Cochrane review, procalcitonin-guided initiation and duration fixing of antibiotic treatment for acute respiratory infections result in reduced mortality, antibiotic use, and antibiotic-related side effects.^54^

Some C-reactive protein assays, particularly commercially available rapid diagnostic tests, could be used in level I care settings to help target antibiotic use in people presenting with febrile or respiratory illness. Diagnostic procalcitonin tests are also commercially available, including a small number of disposable rapid diagnostic tests. Most assays, however, are performed on instrument-based systems designed for use in well resourced clinical laboratories, although a few platforms might be suitable for near–point-of-care testing in LMICs.

In addition to C-reactive protein-based and procalcitonin-based assays, some diagnostic tests that can be used to measure novel biomarkers, combinations of host biomarkers, or combinations of protein biomarkers and gene classifiers are commercially available. Additional host response and biomarker assays remain in the development pipeline.^6^

## Diagnostic pipeline and landscape analysis

Numerous phenotypic and non-phenotypic systems are commercially available for identification, AST, and testing of antibacterial resistance for most pathogens in the BPPL. However, these platforms typically require well equipped laboratories with stable electricity, climate control, running water, appropriate infrastructure, and highly trained personnel, which impedes their feasibility in LMICs.^22^^,^^23^ Therefore, their use is largely restricted to level III or IV laboratories.

Conventional culture-based identification and AST remain central to diagnostic bacteriology, but are inherently slow and labour-intensive, particularly when performed manually.^57^ Culture-based identification might take several days, especially for slow-growing or fastidious organisms.^15^ Automated phenotypic systems reduce the time to result and improve usability, but still require prior culture, which is often unavailable in secondary-level facilities in LMICs. Thus, the acute need for simplified, robust culture solutions tailored for lower-level health-care settings remains.

An increasing number of non-phenotypic commercial platforms enable simultaneous pathogen identification and antibacterial resistance detection. Most platforms, particularly those targeting BSIs, are optimised for well resourced laboratories and skilled personnel. A limited subset might be deployable in near–point-of-care settings, although typically not for culture-dependent BSI testing. Although these platforms provide faster turnaround times than phenotypic methods, they do not replace culture-based identification and AST.

Commercially available phenotypic AST-only platforms are few and are generally unsuitable for level II laboratories and below in LMICs; all require cultured isolates for BSI testing. Similarly, antibacterial resistance-only assays are insufficient in number, typically require instrumentation incompatible with lower-level facilities, and depend on culture for BSI testing. Some assays can detect specific resistance genes directly from urine samples (eg, *E coli* and *Klebsiella* spp).

Several next-generation diagnostic systems that integrate molecular methods with WGS, machine learning, or artificial intelligence are being developed. Some of these systems might eventually be deployable in level II settings. Pipeline platforms vary in scope, with some offering combined pathogen identification and antibacterial resistance detection facilities, whereas others only support monoplex target identification or specific sample types (eg, swabs or urine). Very few systems are designed to process complex samples, including whole blood, which enables direct BSI detection.

In parallel, host-response biomarker assays, such as those for C-reactive protein and procalcitonin, can support infection assessment. Rapid diagnostic tests based on C-reactive protein are already used at level I centres in LMICs, whereas procalcitonin testing remains restricted to a small number of instrument-based platforms suitable for level II settings. Although these inflammatory markers do not provide definitive bacterial pathogen detection, they can support triage, inform assessment of infection severity, and guide initial antimicrobial treatment and de-escalation. Further studies are needed to optimise clinical application, particularly in LMICs.

Finally, novel biomarker platforms, some commercially available and others in development, aim to differentiate viral from bacterial causes of acute respiratory infections at level I care. Although these tools might support antibiotic stewardship, most remain under-evaluated, particularly in LMICs, where robust clinical performance data are limited.^11^^,^^47^^,^^54^

## Diagnostic gaps and needs

Many commercially available systems for bacterial pathogen detection and identification and for AST or antibacterial resistance testing are unsuitable for use across all levels of health-care facilities. Most systems require sophisticated, well equipped laboratories and highly trained laboratory personnel. In LMICs and other low-resource settings, this effectively restricts the availability of these tests to level III and IV health-care facilities. Overall, simpler, faster, and less costly methods for bacterial pathogen identification and AST or antibacterial resistance testing are needed, especially in LMICs.

Substantial technology gaps remain in tests and testing platforms suitable for level I and level II health-care facilities in LMICs. The diagnostic gaps for pathogens in the BPPL, identified through landscape analysis and expert consultation, are summarised in panel 1.

Panel 1 **Identified gaps in diagnostics for priority bacterial pathogens in low-resource settings**

Insufficient simple and easy-to-use tests (for example, host biomarker tests) with good sensitivity and specificity across different population groups to determine whether a patient needs antibiotics, especially at level I health-care facilities

Insufficient simple and easy-to-use tests and platforms that use whole blood or other sample matrices, which need not be cultured (eg, urine or respiratory specimens) and can be tested for antibiotic susceptibility or antibacterial resistance at primary and secondary health-care settings

No multiplex platform to detect bacterial pathogens, including those causing blood-stream infections, from whole blood (not requiring culture), with provision for antibiotic susceptibility or antibacterial resistance testing to be performed on a separate or the same platform, and suitable for use at level I or II facilities

Very limited diagnostic capacity to perform blood culture and antimicrobial susceptibility testing in the context of blood-stream infections, particularly sepsis, at secondary health-care facilities in low-income and middle-income countries

In addition to the identified technology gaps, clinical microbiology laboratories in LMICs require increased human capacity, including well-trained technicians, and improved infrastructure, such as adequate space, uninterrupted electricity, climate control, water supply, sample transport, and strengthened supply chains. Laboratories also require robust quality systems, including written policies and procedures, internal and external quality assurance, and adequate record systems. In the absence of these elements, techniques such as bacterial culture, microscopy and macroscopy, biochemical testing, and most molecular methods are generally available only at level III or IV facilities in LMICs, thus impeding access to testing for most populations.

Advanced automated instrumentation depends on complex medical infrastructure, including extensive sample transportation networks (eg, to collect samples from hospitals and clinics) and sophisticated tracking systems to ensure timely result delivery. Such systems are difficult to adapt for use in most LMICs, where barriers related to access, cost, infrastructure, and loss to follow-up constrain case detection and restrict automated testing to level III and IV laboratories.

Overall, both new and existing technologies should be integrated into appropriate stewardship programmes and policies to ensure correct use and interpretation.

## Research and development priorities

The diagnostic gaps and needs related to pathogens in the BPPL indicate research and development priorities for health-care facilities in low-resource settings, particularly in LMICs, and inform suggested actions for the global health community over the next 3–5 years (panels 2 and 3).

Panel 2 **Research and development priorities based on the landscape analysis of diagnostics and identified gaps**

**Easy-to-use tests to guide antibiotic use**

Rapid diagnostic tests to reliably distinguish bacterial and non-bacterial infections at level I health-care settings are not available. This requirement is often referred to as the holy grail. A test that could be used to identify whether a person has been infected and assess the severity of the infection would also be valuable. Current rapid diagnostic tests based on host biomarkers, such as C-reactive protein and procalcitonin, or multibiomarker combinations, show promise in differentiating bacterial from viral infections and in screening for infection. With adequate performance, these tests could help triage patients with fever at level I health-care facilities. A consensus target product profile for such a test, developed through a Delphi process, was published in 2016.^58^

**Simple and easy-to-use antibiotic susceptibility-only assays**

Although several phenotypic antibiotic susceptibility test-only platforms are commercially available or in development, none of them is designed for use in level II health-care settings or lower. Hence, we currently require cultured samples for antibiotic susceptibility testing (AST) of blood-stream infections (BSIs). For example, a simple, visual, culture-based system providing susceptibility interpretation via colour-coded patterns could be valuable in low-resource settings, where trained laboratory technicians are not available. Ideally, such platforms should enable AST directly from samples such as urine, stool, and nasal swabs, and, optimally, from whole blood. WHO published a target product profile for such an assay in 2020.^59^

**Simple and easy-to-use antibacterial resistance-only assays**

Existing antibacterial resistance-only assays, most requiring instrumentation, are generally unsuitable for level II laboratories or lower, and all such assays currently require cultured samples for BSI testing. Beyond current urine-based assays, development should target platforms capable of performing antibacterial resistance testing directly from samples such as stool and nasal swabs, and, optimally, from whole blood.

**Multiplex diagnostic platforms for bacterial identification and, ideally, antibiotic susceptibility and antibacterial resistance testing without culture**

No multiplex platforms currently exist for use at level II laboratories to directly identify BSIs from whole blood without culture. An optimal system should detect *Escherichia coli*, *Pseudomonas aeruginosa*, *Klebsiella pneumoniae*, *Enterobacter* spp, meticillin-resistant *Staphylococcus aureus,* and *Enterococcus* spp on a single panel and also perform antibiotic susceptibility or antibacterial resistance testing. Although some multiplex systems can identify respiratory or gastrointestinal pathogens from swabs and some others can detect resistance markers from swabs, culture remains necessary for BSI diagnosis. Although a few pipeline-stage platforms aim to integrate identification and antibiotic susceptibility or antibacterial resistance testing, none is yet available. WHO published a target product profile for such a platform in 2020.^59^

**Simplified blood culture for use in level II laboratories**

Blood culture is generally only performed at level III and level IV laboratories in low-income and middle-income countries (LMICs). Blood culture systems for identification of blood stream infections need to be simplified for use in level II facilities in LMICs in order to support patient management and surveillance.^60^ In addition, a sample-in, result-out platform capable of identifying a broad range of bacterial pathogens directly from whole blood or other sample types (eg, urine, stool, or nasal swabs), ideally with integrated antibiotic susceptibility or resistance testing, would be valuable for both level II and III laboratories in LMICs.

**Product innovation and adaptation of existing diagnostics**

Numerous commercial and pipeline diagnostics exist for bacterial pathogen detection, identification, and antibiotic susceptibility or antibacterial resistance testing. However, most of these diagnostics are suitable only for level III or IV laboratories. These systems could be adapted for potential use at level I or II facilities, particularly in LMICs. Potential improvements include eliminating culture requirements, reducing instrument complexity and size, enhancing robustness for challenging environments, and reducing costs.

Panel 3 **Suggested action steps for the global health community to address research and development priorities over the next 3–5 years**

• Evaluate whether the existing biomarker target product profile should be updated. If not, raise awareness among stakeholders regarding the current target product profile and the need for validated host-response tests in primary health care, especially in areas with high co-infection burden. One possible pathway is to identify a biomarker or test with confirmed accuracy (potentially validated in high-income countries) and adapt the technology for use in low-income and middle-income countries (LMICs).
• Although promising and rapid phenotypic antibiotic susceptibility assays are being developed, further research and development are needed to create easy-to-use, interpretable systems capable of delivering complete antibiotic susceptibility test results within hours following pathogen identification. Additional clinical studies are also required to validate novel antibiotic susceptibility systems in the pipeline.
• Encourage research and development to develop simple, affordable antibacterial resistance assays suitable for LMICs and capable of direct testing from diverse clinical samples, following pathogen identification.
• Support research and development to simplify bacterial culture techniques, develop integrated platforms for identification and antibiotic susceptibility or antibacterial resistance testing of priority bacterial pathogens without requiring culture, and develop rapid and affordable assays to detect the presence of microorganisms causing severe infections, such as blood-stream infections, directly from blood samples. Additionally, promote research to simplify existing diagnostics for lower-level settings, informed by user feedback through surveys or similar mechanisms to improve usability, accessibility, and affordability.

## Conclusion

This Review highlights gaps and needs in diagnostics for the detection, identification, and AST or antibacterial resistance testing of pathogens in the WHO BPPL, with emphasis on level I and II health-care facilities in LMICs. The Review concludes that current research and development is insufficient, particularly in addressing diagnostic needs in LMICs. The Review underscores the urgent need for new diagnostics that are user-friendly, rapid, robust, affordable, and accessible to improve clinical practice, support antibiotic stewardship, and ultimately improve patient outcomes.

Research and development efforts to develop and deliver in-vitro diagnostics, including basic research, proof of concept, platform or prototype development, product optimisation, premarket validation, clinical trials, manufacturing scale-up, and commercial release, should proceed alongside diagnostic capacity building. Capacity building includes improved infrastructure, workforce training, and systems development. Governments, stakeholders, and research and development partners should support research that addresses diagnostic gaps and identified development priorities to ensure access to these novel diagnostic tools for patients who need them, particularly in LMICs.

## Declaration of interests

MM and TRoc are consultants to the WHO antimicrobial resistance (AMR) department. TTB has served as a consultant and participated in scientific advisory boards of Sefunda, Combating Antibiotic-Resistant Bacteria Biopharmaceutical Accelerator (CARB-X)/Mod, Biosparq, and the Global AMR Research and Development Hub. BWT works at CARB-X and has participated in scientific advisory boards for the Right Foundation, Pathways to Antimicrobial Clinical Efficacy, and Nesta. BWT has received travel grants from Fondation Mérieux and ValueDx via IMI. The content of this Review is solely the responsibility of the authors and does not necessarily represent the official views of CARB-X or any CARB-X funders. SMP has been a member of bioMérieux advisory board panel and received research support from bioMérieux and Seegene. JV served as a consultant for QuantumMDx Group and Roche and receives research support from QuantumMDx. The other authors declare no competing interests.

## References

[1] WHO (Nov 21, 2023). Antimicrobial resistance. https://www.who.int/news-room/fact-sheets/detail/antimicrobial-resistance.

[2] GBD 2021 Antimicrobial Resistance Collaborators (2024). Global burden of bacterial antimicrobial resistance 1990–2021: a systematic analysis with forecasts to 2050. Lancet.

[3] Nuffield Department of Medicine University of Oxford. Global research on antimicrobial resistance (GRAM). https://www.tropicalmedicine.ox.ac.uk/gram.

[4] UN General Assembly (Sept 9, 2024). Political declaration of the high-level meeting on antimicrobial resistance. https://www.un.org/pga/wp-content/uploads/sites/108/2024/09/FINAL-Text-AMR-to-PGA.pdf.

[5] WHO (May 17, 2024). WHO bacterial priority pathogens list, 2024: bacterial pathogens of public health importance to guide research, development and strategies to prevent and control antimicrobial resistance. https://www.who.int/publications/i/item/9789240093461.

[6] WHO (2025). https://iris.who.int/server/api/core/bitstreams/90c53c14-baef-4c05-9d7b-4e6e74108008/content.

[7] Unitaid (2022). Landscape of innovative tools and delivery strategies for eliminating vertical transmission of HIV, syphilis, hepatitis B, and Chagas in endemic areas. https://unitaid.org/uploads/Landscape-of-innovative-tools-and-delivery-strategies-2022.pdf.

[8] Unitaid (2024). Gonorrhea point-of-care diagnostics technology and market landscape report. https://unitaid.org/uploads/Gonorrhea-point-of-care-diagnostics-technology-and-market-landscape.pdf.

[9] Melendez JH, Edwards VL, Muniz Tirado A (2024). Local emergence and global evolution of *Neisseria gonorrhoeae* with high-level resistance to azithromycin. Antimicrob Agents Chemother.

[10] (Jan 22–24, 2008). Consultation on Technical and Operational Recommendations for Clinical Laboratory Testing Harmonization and Standardization. Helping to expand sustainable quality testing to improve the care and treatment of people infected and affected by HIV/AIDS, TB, and malaria. https://docslib.org/doc/3087932/consultation-on-technical-and-operational-recommendations-for-clinical-laboratory-testing-harmonization-and-standardization.

[11] WHO Health products policy and standards: selection, access and use of in vitro diagnostics. https://www.who.int/teams/health-product-policy-and-standards/assistive-and-medical-technology/medical-devices/selection-access-and-use-in-vitro.

[12] Perry JD (2017). A decade of development of chromogenic culture media for clinical microbiology in an era of molecular diagnostics. Clin Microbiol Rev.

[13] Opota O, Croxatto A, Prod'hom G, Greub G (2015). Blood culture-based diagnosis of bacteraemia: state of the art. Clin Microbiol Infect.

[14] Peri AM, Harris PNA, Paterson DL (2022). Culture-independent detection systems for bloodstream infection. Clin Microbiol Infect.

[15] Gerace E, Mancuso G, Midiri A, Poidomani S, Zummo S, Biondo C (2022). Recent advances in the use of molecular methods for the diagnosis of bacterial infections. Pathogens.

[16] Hattori H, Maeda M, Nagatomo Y (2018). Epidemiology and risk factors for mortality in bloodstream infections: a single-center retrospective study in Japan. Am J Infect Control.

[17] Tille PM (2021).

[18] Bisen PS, Debnath M, Prasad GBKS (2012). Microbes: concepts and applications.

[19] Mohd Hanafiah K, Arifin N, Bustami Y, Noordin R, Garcia M, Anderson D (2017). Development of multiplexed infectious disease lateral flow assays: challenges and opportunities. Diagnostics (Basel).

[20] Ellington AA, Kullo IJ, Bailey KR, Klee GG (2010). Antibody-based protein multiplex platforms: technical and operational challenges. Clin Chem.

[21] St John A, Price CP (2014). Existing and emerging technologies for point-of-care testing. Clin Biochem Rev.

[22] Weile J, Knabbe C (2009). Current applications and future trends of molecular diagnostics in clinical bacteriology. Anal Bioanal Chem.

[23] Frickmann H, Zautner AE, Moter A (2017). Fluorescence in situ hybridization (FISH) in the microbiological diagnostic routine laboratory: a review. Crit Rev Microbiol.

[24] Sibley CD, Peirano G, Church DL (2012). Molecular methods for pathogen and microbial community detection and characterization: current and potential application in diagnostic microbiology. Infect Genet Evol.

[25] Tsalik EL, Bonomo RA, Fowler VG (2018). New molecular diagnostic approaches to bacterial infections and antibacterial resistance. Annu Rev Med.

[26] Wang Y, Lindsley K, Bleak TC (2025). Performance of molecular tests for diagnosis of bloodstream infections in the clinical setting: a systematic literature review and meta-analysis. Clin Microbiol Infect.

[27] Si Y, Zhang T, Chen N (2021). A LAMP-based system for rapid detection of eight common pathogens causing lower respiratory tract infections. J Microbiol Methods.

[28] Chang LJ, Hsiao CJ, Chen B (2021). Accuracy and comparison of two rapid multiplex PCR tests for gastroenteritis pathogens: a systematic review and meta-analysis. BMJ Open Gastroenterol.

[29] Nafea AM, Wang Y, Wang D (2024). Application of next-generation sequencing to identify different pathogens. Front Microbiol.

[30] Hilt EE, Ferrieri P (2022). Next generation and other sequencing technologies in diagnostic microbiology and infectious diseases. Genes (Basel).

[31] Hu T, Chitnis N, Monos D, Dinh A (2021). Next-generation sequencing technologies: an overview. Hum Immunol.

[32] Singhal N, Kumar M, Kanaujia PK, Virdi JS (2015). MALDI-TOF mass spectrometry: an emerging technology for microbial identification and diagnosis. Front Microbiol.

[33] Maurer FP, Christner M, Hentschke M, Rohde H (2017). Advances in rapid identification and susceptibility testing of bacteria in the clinical microbiology laboratory: implications for patient care and antimicrobial stewardship programs. Infect Dis Rep.

[34] Edmiston CE, Garcia R, Barnden M, DeBaun B, Johnson HB (2018). Rapid diagnostics for bloodstream infections: a primer for infection preventionists. Am J Infect Control.

[35] Kassem A, Abbas L, Coutinho O (2023). Applications of Fourier transform-infrared spectroscopy in microbial cell biology and environmental microbiology: advances, challenges, and future perspectives. Front Microbiol.

[36] Gajic I, Kabic J, Kekic D (2022). Antimicrobial susceptibility testing: a comprehensive review of currently used methods. Antibiotics (Basel).

[37] Andrews JM (2001). Determination of minimum inhibitory concentrations. J Antimicrob Chemother.

[38] Kowalska-Krochmal B, Dudek-Wicher R (2021). The minimum inhibitory concentration of antibiotics: methods, interpretation, clinical relevance. Pathogens.

[39] Humphries RM (2020). Update on susceptibility testing: genotypic and phenotypic methods. Clin Lab Med.

[40] Kahlmeter G, Giske CG, Kirn TJ, Sharp SE (2019). Point-counterpoint: differences between the European committee on antimicrobial susceptibility testing and Clinical and Laboratory Standards Institute recommendations for reporting antimicrobial susceptibility results. J Clin Microbiol.

[41] Syal K, Mo M, Yu H (2017). Current and emerging techniques for antibiotic susceptibility tests. Theranostics.

[42] Salam MA, Al-Amin MY, Pawar JS, Akhter N, Lucy IB (2023). Conventional methods and future trends in antimicrobial susceptibility testing. Saudi J Biol Sci.

[43] Atsbaha AH, Gedla DG, Shfare MT (2017). Phenotypic tests of bacterial antimicrobial susceptibility testing: a systematic review. SM J Clin Med.

[44] Wenzler E, Maximos M, Asempa TE, Biehle L, Schuetz AN, Hirsch EB (2023). Antimicrobial susceptibility testing: an updated primer for clinicians in the era of antimicrobial resistance: insights from the Society of Infectious Diseases Pharmacists. Pharmacotherapy.

[45] Zhang J, Yang F, Sun Z (2022). Rapid and precise identification of bloodstream infections using a pre-treatment protocol combined with high-throughput multiplex genetic detection system. BMC Infect Dis.

[46] Yusa T, Tateda K, Ohara A, Miyazaki S (2017). New possible biomarkers for diagnosis of infections and diagnostic distinction between bacterial and viral infections in children. J Infect Chemother.

[47] Aabenhus R, Jensen JUS, Jørgensen KJ, Hróbjartsson A, Bjerrum L (2014). Biomarkers as point-of-care tests to guide prescription of antibiotics in patients with acute respiratory infections in primary care. Cochrane Database Syst Rev.

[48] Melbye H, Francis NA, Butler CC (2011). Inflammatory markers are helpful when treating LRTI in primary care. Prim Care Respir J.

[49] Kapasi AJ, Dittrich S, González IJ, Rodwell TC (2016). Host biomarkers for distinguishing bacterial from non-bacterial causes of acute febrile illness: a comprehensive review. PLoS One.

[50] Isaeva E, Akylbekov A, Bloch J (2024). The feasibility of C-reactive protein point-of-care testing to reduce overuse of antibiotics in children with acute respiratory tract infections in rural Kyrgyzstan: a pilot study. Pediatr Health Med Ther.

[51] Espy MJ, Uhl JR, Sloan LM (2006). Real-time PCR in clinical microbiology: applications for routine laboratory testing. Clin Microbiol Rev.

[52] Lubell Y, Blacksell SD, Dunachie S (2015). Performance of C-reactive protein and procalcitonin to distinguish viral from bacterial and malarial causes of fever in Southeast Asia. BMC Infect Dis.

[53] Cooke J, Butler C, Hopstaken R (2015). Narrative review of primary care point-of-care testing (POCT) and antibacterial use in respiratory tract infection (RTI). BMJ Open Respir Res.

[54] Schuetz P, Wirz Y, Sager R (2017). Procalcitonin to initiate or discontinue antibiotics in acute respiratory tract infections. Cochrane Database Syst Rev.

[55] Sager R, Kutz A, Mueller B, Schuetz P (2017). Procalcitonin-guided diagnosis and antibiotic stewardship revisited. BMC Med.

[56] Rhee C (2016). Using procalcitonin to guide antibiotic therapy. Open Forum Infect Dis.

[57] Cohen J, Vincent JL, Adhikari NKJ (2015). Sepsis: a roadmap for future research. Lancet Infect Dis.

[58] Dittrich S, Tadesse BT, Moussy F (2016). Target product profile for a diagnostic assay to differentiate between bacterial and non-bacterial infections and reduce antimicrobial overuse in resource-limited settings: an expert consensus. PLoS One.

[59] WHO (Feb 19, 2019). Target product profiles for antibacterial resistance diagnostics. https://www.who.int/publications/i/item/10665331054.

[60] Dailey PJ, Osborn J, Ashley EA (2019). Defining system requirements for simplified blood culture to enable widespread use in resource-limited settings. Diagnostics (Basel).

